# The introduction history of invasive garden ants in Europe: Integrating genetic, chemical and behavioural approaches

**DOI:** 10.1186/1741-7007-6-11

**Published:** 2008-02-26

**Authors:** Line V Ugelvig, Falko P Drijfhout, Daniel JC Kronauer, Jacobus J Boomsma, Jes S Pedersen, Sylvia Cremer

**Affiliations:** 1 Centre for Social Evolution, Department of Biology, University of Copenhagen, Universitetsparken 15, 2100 Copenhagen, Denmark; 2 Evolution, Genetics and Behaviour, Biology I, Institute of Zoology, University of Regensburg, 93040 Regensburg, Germany; 3 Chemical Ecology, School of Physical and Geographical Sciences, Keele University, Keele, Staffordshire, ST5 5BG, UK

## Abstract

**Background:**

The invasive garden ant, *Lasius neglectus*, is the most recently detected pest ant and the first known invasive ant able to become established and thrive in the temperate regions of Eurasia. In this study, we aim to reconstruct the invasion history of this ant in Europe analysing 14 populations with three complementary approaches: genetic microsatellite analysis, chemical analysis of cuticular hydrocarbon profiles and behavioural observations of aggression behaviour. We evaluate the relative informative power of the three methodological approaches and estimate both the number of independent introduction events from a yet unknown native range somewhere in the Black Sea area, and the invasive potential of the existing introduced populations.

**Results:**

Three clusters of genetically similar populations were detected, and all but one population had a similar chemical profile. Aggression between populations could be predicted from their genetic and chemical distance, and two major clusters of non-aggressive groups of populations were found. However, populations of *L. neglectus *did not separate into clear supercolonial associations, as is typical for other invasive ants.

**Conclusion:**

The three methodological approaches gave consistent and complementary results. All joint evidence supports the inference that the 14 introduced populations of *L. neglectus *in Europe likely arose from only very few independent introductions from the native range, and that new infestations were typically started through introductions from other invasive populations. This indicates that existing introduced populations have a very high invasive potential when the ants are inadvertently spread by human transport.

## Background

Exotic species are a major threat to biodiversity and human welfare, and infestations by invasive species have generally proven difficult and expensive to exterminate [1-3]. The cheapest and most effective control strategy would thus be to prevent invasive species from infesting new localities from the beginning [4]. This is only possible, however, if detailed knowledge of the pathways of introduction into new localities is available [5-7]. This type of information is difficult to obtain by direct monitoring, as today most invasive species are introduced unintentionally by human activities: one example is the transport of ballast water, by which ships regularly mix marine species across continents, creating an enormous potential for species invasions [8]. Furthermore, population sizes of introduced species are typically small and thus unrecognisable on arrival, therefore only becoming obvious after lag phases that can reach up to several decades [9]. An increasing number of studies have thus used genetic markers in an attempt to trace back source populations and estimate the number of independent introduction events [10-13], as in the case of an avocado-infecting thrips species recently introduced to California that is now an agricultural pest [11]. Genetic marker studies are of great general significance as they can be used to not only unravel phylogeographic relationships between populations, but also to estimate the genetic diversity within populations, which typically reflects the severity of founder effects in each population (genetic bottlenecks) [14,15]. These genetic effects should be even more pronounced in populations that are a result of sequential introduction events (see, for example, [16]).

Invasive species are not limited to specific taxa or habitats and range from terrestrial plants such as the fire tree (*Myrica faya*) to aquatic animals such as the zebra mussel (*Dreissena polymorpha*) [17]. Social insects, particularly ants, seem to be especially efficient invaders, with the ants representing 5 species of the list of "100 of the world's worst invasive alien species" [17]. The best known invasive ants are the red imported fire ant *Solenopsis invicta*, the Argentine ant *Linepithema humile *and the Pharaoh ant *Monomorium pharaonis *[18]. However, with the exception of invasive Pharaoh ants that inhabit buildings, pest ants have so far remained restricted to climates with warm winters and have not been able to penetrate any cold-temperate regions.

This picture changed in 1990 when the invasive garden ant *Lasius neglectus *was described in Hungary [19]. *L. neglectus *is well adapted to cold winters and is currently spreading quickly throughout Europe and Asia with 30 populations known in 2000 and 100 populations identified to date [20]. It has been estimated that the invasive garden ant can survive mean winter temperatures down to -5°C (see [21]), which indicates that it has not even come close to reaching the limits of its potential distribution: from Southern Sweden and Scotland in the West to China and Japan in the East [21,22]. *L. neglectus *typically occurs in human-disturbed urban habitats such as parks, greenhouses and gardens [19] and has so far not been found in any natural habitat. Partly because of this, its native range is not yet known, but the Black Sea area is the most likely candidate [21].

Similar to other invasive ants [18,23], *L. neglectus *forms large networks of interconnected and mutually tolerant nests, each of which contains multiple queens [24]. This 'supercolonial' population structure differs from the social organisation of native ant species, which typically show high aggression against neighbouring nests [25]. Supercoloniality is an important factor contributing to the ecological dominance of invasive ants as it allows very high nest densities, which greatly benefits foraging efficiency [26]. In addition to this exploitation competition advantage, invasive ants are often highly aggressive towards native ants [27-29], and this is also true for *L. neglectus *[30]. It is therefore not surprising that the invasive garden ant normally out-competes native ant species and eliminates most other arthropods where it has been introduced [31,32].

Invasive ants have generally lost the typical mating flights that characterise most ants: they mate inside the nest and found new colonies by nest-budding [18,23]. For long distance dispersal, they rely on human mediated transport, which often occurs when soil and plant material is moved from an infested site to another area. Newly founded populations that share the same origin are thus expected to be genetically similar and mutually non-aggressive, but genetic markers and aggression are not necessarily tightly correlated with geographic distance because human transport may often result in 'jump dispersal' over long distances [33]. Supercolonies of invasive ants can therefore span mutually tolerant populations that are geographically separated but all derived from the same introduction. The largest supercolony to date has been described for the Argentine ant in Southern Europe and spans populations as far apart as 6000 km [34].

The geographic extension of invasive ant supercolonies (either continuous or disjunctive) is widely used to reconstruct their invasion history [35] and represents an additional source of behavioural information not available in studies of invasive species in general. Recognition and aggression behaviour are based on the expression of cuticular hydrocarbon profiles, which represent the recognition cues of social insects [34,36-38]. In this study, we therefore combine genetic and behavioural analyses with chemical analysis of cuticular hydrocarbon profiles to estimate supercolony expansion of the invasive garden ant. Our aim is to evaluate the efficiency of these three different approaches to detect supercolony boundaries and to test whether they give consistent information for reconstructing the invasion history of *L. neglectus *in Europe. We also try to obtain an estimate of how many independent introductions from the native range might have occurred, and how often new populations might have arisen from already existing invasive populations. This type of information is pertinent in the development of efficient biocontrol strategies against the further spread of the invasive garden ant.

## Results

### Size and range of supercolonies

#### Estimates from genetic analysis

**Figure 1:**
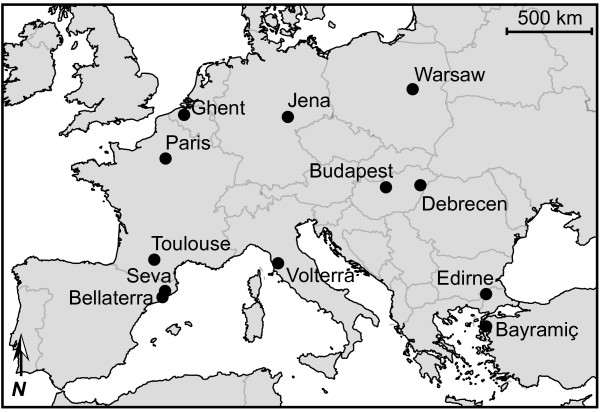
**Study populations of *L. neglectus***. The 14 sampled populations in cities across Europe (three populations in Edirne).

**Figure 2:**
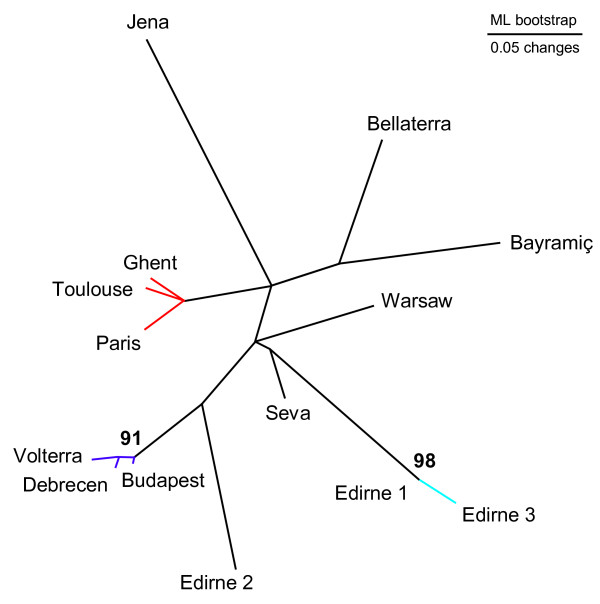
**Phylogeny of the European invasive *L. neglectus *populations**. Unrooted best tree (six microsatellite loci), using Cavalli-Sforza chord measures (bootstraps over 50% are indicated). Coloured branches indicate three clusters that were independently identified as highly similar in Bayesian clustering analysis (BAPS program).

As expected for unicolonial ant populations [25,39], the study populations (Figure 1) were not genetically substructured at the nest level so that the five sampled nests per population represent a random sample of the overall genetic composition of the local supercolony (mean regression relatedness, 0.015; 95% confidence interval, -0.028–0.057). We therefore used 30 individuals per population as independent samples when performing our between-population analyses and did not consider the nest level any further. The overall genetic differentiation among populations (*F*_ST_) was on average 0.334 (with pairwise estimates ranging from 0.249 to 0.431; 87 of the 91 pairwise estimates were significant; see Additional file 1). The six studied microsatellite loci thus carry enough information to differentiate between populations in spite of their moderate polymorphism. This result remains unchanged and significant when omitting any of the six loci. Phylogenetic analysis of the microsatellite data revealed two clusters with two and three populations of high genetic similarity (Figure 2). These were, first, two of the three populations within Edirne, Turkey (Edirne 1 and Edirne 3; bootstrap value 98%) and, second, the two populations in Hungary (Budapest and Debrecen) and the population from Italy (Volterra; bootstrap value 91%). Additional Bayesian clustering analysis supported these two clusters and also grouped the populations in France (Paris and Toulouse) and Belgium (Ghent; posterior probability for this partitioning to be correct of over 99%). The genetic analysis therefore revealed three genetically different but internally similar clusters of populations, whereas six populations could not be consistently clustered into genetic groups (overall *F*_ST _between Bayesian Analysis of Population Structure (BAPS) clusters and populations not assigned to clusters was 0.345; pairwise values ranging from 0.108 to 0.551, all significant).

#### Estimates from chemical analysis

**Figure 3:**
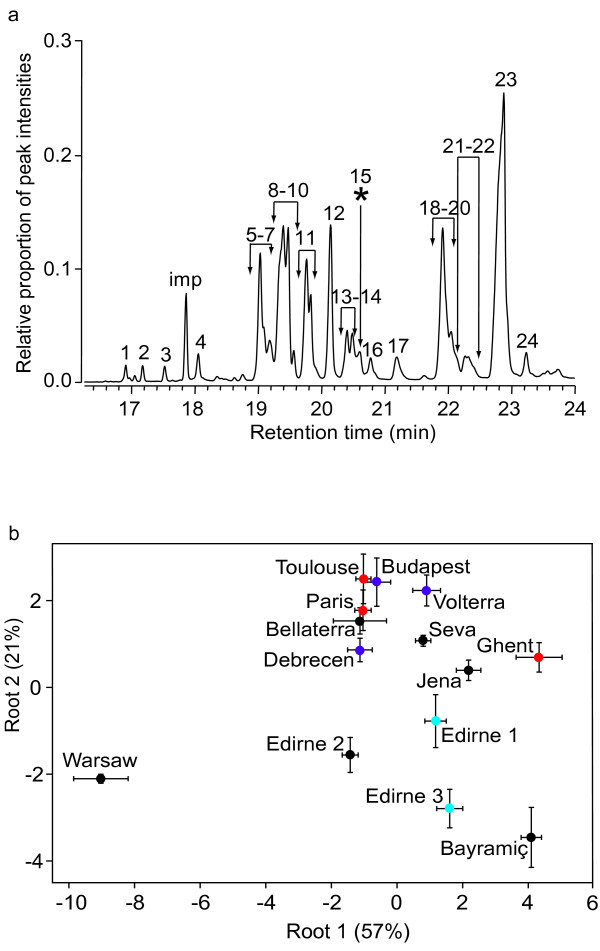
**Cuticular hydrocarbon patterns of *L. neglectus***. (a) Cuticular hydrocarbon profile of *L. neglectus*, consisting of 24 hydrocarbon peaks. Hydrocarbon peak 15* was only found in the Warsaw population. Peak numbers correspond to the following compounds: **1**, 11C31:1; **2**, C31; **3**, 13MeC31; **4**, 3MeC31; **5**, C33:2; **6**, C33:2; **7**, C33:2; **8**, 12C33:1 + 13C33:1; **9**, 10C33:1 + 11C33:1; **10**, 7C33:1; **11**, 13Me21C33:1 + 13Me23C33:1; **12**, 13MeC33 + 15MeC33; **13**, 3Me21C33:1; **14**, 3Me23C33:1; 15*, 10,23diMeC33; **16**, 3MeC33 + 5,15diMeC33; **17**, 12,14,22Me21C34:1 + 12,14,22Me23C34:1; **18**, C35:2; **19**, C35:2; **20**, C35:2; **21**, 21C35:1; **22**, 23C35:1; **23**, 13,15Me21C35:1 + 13,15Me23C35:1; **24**, 13MeC35 + 15MeC35 ('imp' denotes impurity). (b) Discriminant analysis of all 14 populations (mean and standard error of three to seven nests per population), with the first and second extracted root (and the variance they explain). Populations are coloured according to the genetic clusters (Figure 2).

Gas chromatography-mass spectrometry (GC-MS) analysis of cuticular compounds produced a total of 24 different hydrocarbons, of which 23 were consistently found in each population, and with the remaining single compound (peak 15*) only present in the population from Warsaw, Poland (Figure 3a; for details see Additional file 2). Discriminant analysis based on the 8 factors extracted from the 24 hydrocarbons in a principal component analysis (PCA; explaining 82% of the total variance) and using population as a grouping variable, showed that the multiple nests analysed per population always clustered very closely and that populations were distinct (Figure 3b; Wilks' lambda: 0.00058; *F*_104,334 _= 6.27; *P *< 0.0001). The Warsaw population with the additional single hydrocarbon peak 15* was considerably separated from all other populations, so that all nests of this population had a 100% posterior probability to be correctly assigned to this particular population based on their chemical profiles. In the remaining populations, correct assignments were in the range 35–97%, with a total of 11 out of 68 nests misclassified; see Additional file 3 for details). To test whether the considerable difference between Warsaw and the other populations might have masked differences among the remaining populations, we repeated the analysis without the Warsaw population. This lead to very similar posterior probabilities of nests being assigned to their original populations in the range 33–94%, with only a slightly higher proportion of nests (13/63) being misclassified.

#### Estimates from analysis of aggression behaviour

**Figure 4:**
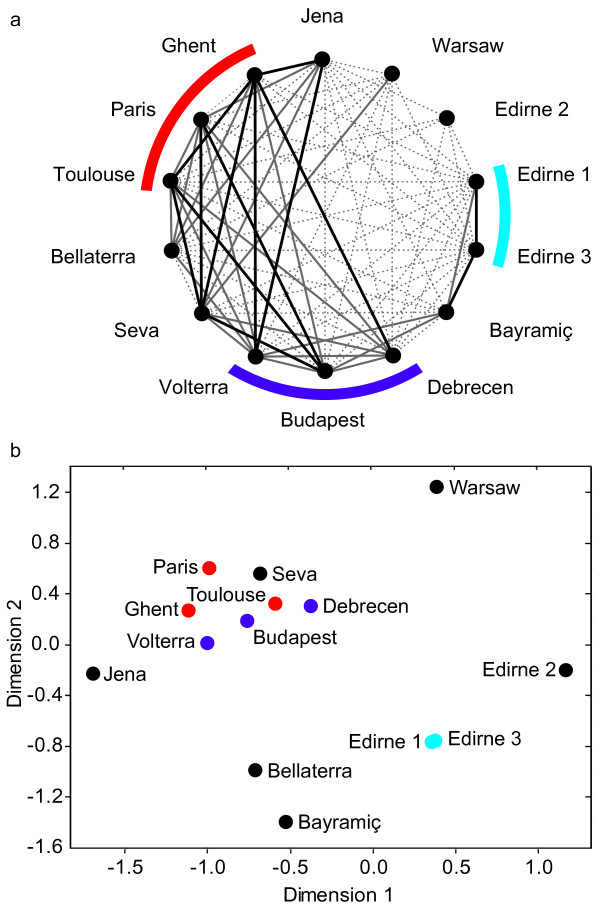
**Aggression behaviour between European *Lasius neglectus *populations**. (a) Population pairs, in which aggression did not occur in any of the replicates, are connected with a black solid line. When aggression occurred in less than 50% of the replicates, population pairs are connected with a solid grey line, and when aggression occurred in more than 50% they are connected with a dotted grey line. Unconnected population pairs were aggressive in all replicates. For comparison, the genetic clusters of Figure 2 are shown in colours. (b) Multidimensional scaling plot representing the behavioural distance between the 14 populations. The plot is based on the proportion of aggressive interactions between population pairs.

Aggression never occurred within nests (*n *= 60) nor between nests of the same population (*n *= 179), confirming our genetic results that populations consisted of single supercolonies. In the between-population tests, aggression was detected in none of the replicates (mean 7) in 14% of the 91 independent pairwise combinations of populations (total *n *= 579 tests), but always occurred in 19% of the combinations. The remaining 67% showed aggression in some replicates, but not in others. However, the variation between population pairs was significantly higher than the variation between replicates (Kruskal-Wallis: χ^2^_81 _= 201; *P *< 0.0001). Based on these data, we generated an 'aggression network' (Figure 4a), connecting only those populations that were always non-aggressive and thus were inferred to be part of the same supercolony with black solid lines. This revealed one supercolony containing the two genetic clusters Belgium-France and Hungary-Italy, and another smaller one, including the Edirne 1+3 cluster. Despite this grouping, the boarders of these behavioural supercolonies remained somewhat blurry, as, for example, the populations in Budapest and Seva and the populations of Seva and Volterra showed no mutual aggression, yet ants from Budapest and Volterra did react aggressively when encountering one another. It is therefore not possible to predict the outcome of encounters between two populations based on their behaviour towards a third party. Three populations were not part of any behavioural supercolony: one of the Spanish populations (Bellaterra), the third population collected in Edirne, Turkey (Edirne 2) and the Polish population in Warsaw. In addition, we created a multidimensional scaling plot (Figure 4b) of the populations based on their 'behavioural distances' (that is, the aggression probabilities of pairwise population encounters; see Additional file 1 for details).

### Correlations between geographic, genetic, chemical and behavioural distance

Partial correlation analysis showed that the geographic and genetic distances between the populations were not significantly associated (Mantel test, *r*_geo,gen_: *P *= 0.44), as expected under a scenario of jump dispersal. Also, the chemical distances between populations were not significantly correlated with geographic (Mantel test, *r*_geo,chem.gen_: *P *= 0.34) or genetic distances (Mantel test, *r*_gen,chem.geo_: *P *= 0.48). However, the behavioural distance between populations, that is, the pairwise aggression level, was significantly correlated with geographic (*r*_geo,behav.gen.chem _= 0.296, *P *= 0.009), chemical (*r*_chem,behav.gen.geo _= 0.353, *P *= 0.003) and genetic distance between populations (*r*_gen,behav.chem.geo _= 0.375, *P *= 0.001). As it turned out, aggression was lower when populations were geographically closer, chemically more similar and genetically more related (see Additional file 1 for plots).

### Age and diversity of populations

**Figure 5:**
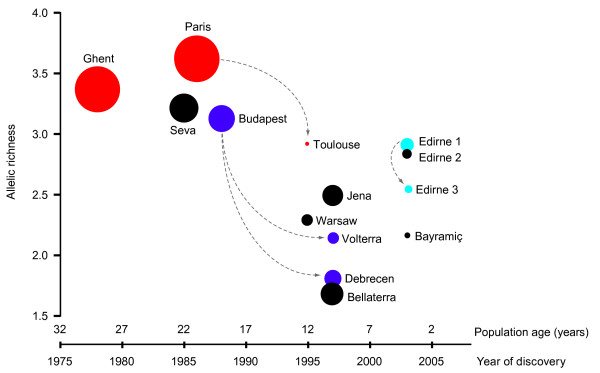
**Genetic diversity as a function of population age**. Mean allelic richness as a function of population age and population size (size of dots proportional to population area as given in Table 1) showing that allelic richness is generally lower in younger and smaller populations of *L. neglectus*. Arrows indicate likely genetic relations within genetic clusters, that is, older and more genetically diverse populations probably have given rise to younger populations. Populations are coloured according to the genetic clusters.

The true age was unknown for most populations, so we used 'year of discovery' as the best possible (minimal) estimate. As all populations were assumed to have started with a small founder group of a single or very few nests, the present size of a population was also expected to be an indicator of age and both were indeed highly correlated (Pearson correlation, *n *= 14, *r *= -0.838, *P *= 0.0002; see Table 1 for details). We determined the chemical variation within populations (that is, the mean chemical distance of nests to their group centroid; Table 1) and found that the chemical variation was significantly negatively correlated with the discovery date of the populations (Pearson correlation, *n *= 14, *r *= -0.513, *P *= 0.060) and positively correlated with present population size (*r *= 0.688, *P *= 0.006). We found similar results for correlations of the allelic richness for each population based on the microsatellite analysis, with both year of detection (Figure 5; Pearson correlation, *n *= 14, *r *= -0.600, *P *= 0.023) and population size (*r *= 0.632, *P *= 0.0154), as well as a high correlation between allelic richness and chemical within-population variation (*r *= 0.745, *P *= 0.002). Also, the number of polymorphic loci (out of the six studied; Table 2) was typically low in the youngest populations, discovered after 1995 (Table 1). Genetic bottlenecks were detected in three populations (Bayramiç, Edirne 3, Jena; Table 1) when using the software M RATIO, but the software BOTTLENECK did not detect any recent decrease in effective population size. Investigating the allelic distributions among populations within the same genetic cluster showed that the smaller Edirne 3 population only contained a fraction of the alleles found in the larger Edirne 1 population (except for two rare alleles, 272 and 292, in L10–174 that were only found in Edirne 3; see Table 3). Likewise the alleles present in the smaller populations in Debrecen and Volterra were subsets of the alleles in the larger Budapest population (with the exception of one rare allele, 284, found in Volterra in L10–174). Similarly, the alleles in the small Toulouse population were a subset of the larger population in Paris (except for two rare alleles, 240 and 274, in L10–174 found in Toulouse). This led us to draw a likely scenario for the history of introductions of the European populations of *L. neglectus *as shown in Figure 5.

**Table 1:** Age and diversity of European *Lasius neglectus *populations. Population name, country and year of discovery; *A*, population size in square kilometres; CHC var, within-population variation of cuticular hydrocarbon profiles; *P*, number of polymorphic loci (out of six microsatellite loci); *k*', average allelic richness across all loci; *DH/sd*, standardised difference between the expected heterozygosity under mutation-drift equilibrium and observed heterozygosity; *M*, ratio between allele number and range. The latter two estimates are averages across polymorphic loci. Bold figures indicate significances in individual tests, whereas asterisks indicate significance levels after sequential Bonferroni correction for multiple tests: *P < 0.05; **P < 0.01.

Population	Year	*A*	CHC var	*P*	*k*' ± SE	*DH/sd *± SE	*M*
Ghent, Belgium	1978	0.57	10.797	4	3.37 ± 1.02	-0.23 ± 0.39	**0.576**
Seva, Spain	1985	0.20	8.413	4	3.21 ± 1.15	-0.23 ± 0.36	0.625
Paris, France	1986	0.57	12.538	5	3.62 ± 1.09	0.45 ± 0.37	0.742
Budapest, Hungary	1988	0.20	4.777	5	3.11 ± 0.77	-1.88 ± 0.71	**0.603**
Toulouse, France	1995	0.01	5.566	3	2.92 ± 0.97	-0.28 ± 0.81	0.560
Warsaw, Poland	1995	0.04	4.691	3	2.29 ± 0.64	0.43 ± 0.35	0.694
Bellaterra, Spain	1997	0.14	3.498	3	1.66 ± 0.33	0.34 ± 0.73	0.756
Debrecen, Hungary	1997	0.08	3.338	2	1.80 ± 0.52	-0.94 ± 1.33	0.563
Jena, Germany	1997	0.14	5.796	5	2.49 ± 0.42	1.06* ± 0.15	0.503*
Volterra, Italy	1997	0.04	3.141	3	2.14 ± 0.58	-0.86 ± 0.43	0.720
Bayramiç, Turkey	2003	0.01	7.730	3	2.16 ± 0.54	0.35 ± 0.20	0.338**
Edirne 1, Turkey	2003	0.05	8.545	4	2.91 ± 0.83	0.19 ± 0.51	0.600
Edirne 2, Turkey	2003	0.03	2.405	3	2.84 ± 0.94	-1.28 ± 1.36	**0.551**
Edirne 3, Turkey	2003	0.02	7.100	3	2.55 ± 0.77	-0.25 ± 0.60	0.419*

**Table 2:** Microsatellite loci used in the study. Name, GenBank accession numbers, repeat motif and primer sequences (F: forward, R: reverse primer) are given for the three newly developed microsatellite loci for *L. neglectus*. For other loci see Fjerdingstad et al [58]. The product size in basepairs (bp) and the optimal annealing temperature in degrees Celsius (°C) are shown for *L. neglectus. N*, number of studied populations of *L. neglectus*; *n*, number of individuals; *k*, observed number of alleles; *H*_o_, observed heterozygosity; *H*_e_, expected heterozygosity assuming no genetic differentiation among populations.

**Locus**	**Accession number**	**Repeat motif**	**Primer sequence (5'-3')**	**Product size (bp)**	**Annealing temperature (°C)**	***N***	***n***	***k***	***H*_o_**	***H*_e_**
Lng-1	[GenBank:EU057967]	(CA)_5_T (AC)_2_AA (CA)_13_	F: TCTCGCTCCAACTACTTAAA R: TTGTCTTCAAATTGTCCAAC	204–220	55	14	417	6	0.37	0.64
Lng-3	[GenBank:EU057968]	(CA)_14_	F: GATGCCAAGTTTACATGG R: CACAATACTCACATATTCACAA	112–132	55	14	417	6	0.06	0.11
Lng-4	[GenBank:EU057969]	(CA)_11_	F: GTGAACGAATATATCATGGAT R: CGTTGACGAAAATAGCTC	159–167	55	14	415	4	0.06	0.11
L1–5	--	--	--	285–301	55	14	402	9	0.56	0.81
L10–174	--	--	--	234–298	62	14	416	20	0.42	0.88
L10–282	--	--	--	256–258	55	14	414	2	0.02	0.02

**Table 3:** Allele distributions in European populations of *L. neglectus*. The name of alleles (given as size in basepairs) and the number of alleles in each sampled population of *L. neglectus*, using the six microsatellite loci (Table 2). *n*, the total number of alleles scored in a population at a given locus. The populations are ordered by geography as in Figure 4.

**Locus**	**Size**		**Jena**	**Warsaw**	**Edirne 2**	**Edirne 1**	**Edirne 3**	**Bayramiç**	**Debrecen**	**Budapest**	**Volterra**	**Seva**	**Bellaterra**	**Ghent**	**Paris**	**Toulouse**
Lng-1		***n***	**60**	**60**	**60**	**60**	**60**	**60**	**58**	**60**	**60**	**58**	**58**	**60**	**60**	**60**
	204		45	22		4	3	13				8		20	26	17
	206				31									2		
	214		15		12									5	9	11
	216					34	44			1						
	218			36	17	22	13	9	58	58	53	50	58	33	25	32
	220			2				38		1	7					

Lng-3		***n***	**60**	**60**	**60**	**60**	**60**	**60**	**56**	**60**	**60**	**58**	**60**	**60**	**60**	**60**
	112		19										22	1		
	114					1										
	116		41	60	60	59	60	60	56	56	60	57	38	59	60	60
	118											1				
	120									2						
	132									2						

Lng-4		***n***	**60**	**60**	**60**	**60**	**58**	**60**	**56**	**60**	**60**	**60**	**60**	**56**	**60**	**60**
	159		19	60	60	60	58	60	56	58	60	60	60	56	57	60
	161														3	
	165		22													
	167		19							2						

L1–5		***n***	**58**	**60**	**60**	**60**	**56**	**60**	**54**	**58**	**50**	**60**	**50**	**60**	**60**	**58**
	285					1		39					17			
	287			3		15	14					14		3		
	289			43	1	22	23	10				18		10	7	3
	291				1	5	1		1	1	3	9	4		5	8
	293		22	14	7				27	31	33	3	29	24	18	32
	295		28		49	2		11	18	17	13	6		10	17	9
	297				2	15	18		8	9	1	2		1	12	6
	299		8									7		12	1	
	301											1				

L10–174		***n***	**60**	**58**	**58**	**60**	**60**	**60**	**58**	**60**	**60**	**60**	**58**	**60**	**60**	**60**
	234									1						
	236								54	41	46	6		1		
	238			12	22	11	2	4	2	7		7		3	5	
	240		18	27				34				29	56			4
	242		29	9						4		7	2	19	16	32
	244			10						2				7	8	5
	248														2	
	272						1									
	274															3
	276					19	33								7	1
	278					3	6	20								
	280					19	17	2		1	2	1		3	4	
	282				1				2	4	10	10		22	13	14
	284		2								2			5	5	1
	286		11		2											
	288				25											
	290				4											
	292				1		1									
	296					8										
	298				3											

L10–282		***n***	**60**	**60**	**58**	**60**	**60**	**60**	**56**	**60**	**60**	**60**	**58**	**56**	**60**	**60**
	256		60	60	58	60	60	60	56	60	60	60	58	56	51	60
	258														9	

## Discussion

Our combinatory approach including genetic, chemical and behavioural data allowed us to evaluate the relative suitability of these different methods, and to (at least in part) reconstruct the likely introduction history of *L. neglectus *in Europe. First of all, we could not detect any aggression between nests of the same population, or any genetic substructure within populations, confirming that our study populations represented single supercolonies. When performing between-population analyses, the phylogenetic tree of the 14 *L. neglectus *populations revealed two clades supported with high bootstrap values, but otherwise remained relatively unresolved despite high variation in the microsatellite loci. This indicates that most introduced populations of *L. neglectus *in Europe are likely of rather recent date and of fairly similar origin. The Bayesian clustering analysis confirmed the two well-supported clades from the microsatellite tree, and detected a third cluster of populations. Together, the genetic data therefore indicate that the populations within these three clusters Belgium-France, Hungary-Italy and Edirne 1+3 share a recent common introduction history.

The chemical analysis separated the population in Warsaw, Poland, from all other populations, a split that was not detected by genetic analysis, but that was confirmed by aggression data (see below). All other populations showed high similarities in their chemical profile, consistent with a possible joint origin or with similar environmental conditions. A comparable degree of chemical uniformity was found by Errard et al. [40] when analysing introduced populations of the invasive ant *Wasmannia auropunctata *in the New Caledonia archipelago.

Overall aggression levels between *L. neglectus *populations were very low in our laboratory experiments, which is consistent with the generally high similarities in the genetic and chemical analyses. Remarkably, the observed aggression patterns could be predicted by the genetic and chemical distances between the populations, as well as by their geographic distance. Although Mantel tests do not directly allow testing of the proportions of variance explained by the different predictor variables, the partial correlation coefficients suggest that genetic distance was the best predictor of aggression, followed by chemical and geographic distances. This is in line with other studies showing that aggression is based on chemical recognition cues encoded in the ants' hydrocarbon profiles [36,41] and suggests that these hydrocarbon profiles have a significant genetic component [42-44]. However, considerable amounts of variation in aggression remained unexplained by the overall genetic distance at the microsatellite marker loci, suggesting that environmental conditions in the habitat and food also likely affected the cuticular hydrocarbon profiles.

Although other invasive ants typically have populations that show a clear affiliation to a specific supercolony [34,40,45], no such clear association could be found in *L. neglectus*. Only three populations, Bellaterra, Edirne 2 and Warsaw, performed aggression in all replicates towards all other populations (Figure 4). As *L. neglectus *shows extremely high aggression in inter-specific aggression tests performed under the same laboratory conditions [30], we assume that our data reflect the natural situation and are not a laboratory artefact.

Similar to other invasive ants [18,46,47] and invasive species in general [48,49], there was no pattern of 'isolation by distance', that is, a positive correlation between genetic and geographic distances among populations. Given the limited dispersal of *L. neglectus *queens, this suggests that jump dispersal via human mediated transport is very likely. This was also suggested by earlier studies on *L. neglectus *linking the local appearance of new populations to the introduction of, for example, potted plants [19,32,50], and is complementary to our findings on regional and continental expansion patterns in *L. neglectus *[19]. Some invasion 'hotspots' could be found in several regions, such as the surroundings of Barcelona and within Budapest, Edirne and Warsaw (N Aktaç, personal communication; see also [[19,20,32,51,52]]). These likely arise because of frequent human transport of ant-infested potted plants or soil between nearby construction sites within regions. However, no *a priori *general inferences could be made on the supercolony affiliation of geographically close populations. Two of the three populations in Edirne, Turkey, were extremely similar, whereas the third was different, and the same was true for the two sites studied in the surroundings of Barcelona, Spain.

It was unexpected that so few populations showed detectable signs of a recent genetic bottleneck, as introduced populations in general show low levels of genetic diversity [15], which is the most powerful method in detecting genetic bottlenecks [53]. However, as this method requires data from native populations in equilibrium as a comparison, which are not available for *L. neglectus*, we chose two software packages that do not require this, but instead rely on polymorphic loci only. The applied methods (*M*-ratio and heterozygosity excess) were recently evaluated by Williamson-Natesan [54], who concluded that the *M*-ratio was most likely to detect bottlenecks when they lasted for several generations, when the population had recovered and when the pre-bottleneck population sizes (θ) were large. The heterozygosity excess method, on the other hand, was better at detecting bottlenecks when they were recent, less severe and when pre-bottleneck population sizes were small. The low number of detected bottlenecks in the present study could be an artefact of our analysis as many of the young populations of the invasive garden ant, however, have few polymorphic loci, which in itself is an independent indication of genetic bottlenecks, but it compromises the detection power of these programs [55]. It is therefore likely that the sensitivity of these methods was not sufficient, and that more populations have gone through a recent genetic bottleneck than the three that we could detect. Alternatively, we see the same pattern as in the study by Clegg et al [16] on silvereye birds colonising islands. They found that a single colonisation event was not followed by severe founder effects, but four to five successive colonisation events were required.

In addition to these well-known and broadly applied methods to detect founder effects based on microsatellite data, we have (as far as we are aware for the first time) tested the suitability of the within-population variation of cuticular hydrocarbons. We found that this chemical variation behaves in a very similar manner to allelic richness and, at least in our study, similarly correlates with both the age and size of populations. Our data showed that the younger populations of *L. neglectus *are characterised by the lowest chemical variation and genetic diversity in terms of allelic richness (strongest evidence), the number of polymorphic loci and also the detection of genetic bottlenecks. These data and the considerable similarity between introduced populations make a high number of independent introductions from the native range unlikely. As the founder populations of all extant invasive populations were apparently rather similar, the patterns observed are best explained by assuming that younger populations tend to arise as daughter populations from older introduced populations, consistent with the allelic subset pattern discussed above. This implies that *L. neglectus *experienced serial genetic bottlenecks, which explains the low genetic diversity of young infestations compared with older invasive populations. The higher genetic diversity in the older populations is unlikely to be a result of accumulation of mutations after population founding, as the differences in age are only a few decades. The total evidence available thus allowed us to tentatively infer the most likely chains of ancestry across the investigated 14 populations (Figure 5) as a working hypothesis for further research on the invasion history of *L. neglectus *in Europe. The prevailing pattern is that secondary infestations both have a later discovery date and a smaller population size.

## Conclusion

To the best of the authors' knowledge, this is the first study to simultaneously implement genetic, chemical and behavioural methodology to assess the affiliation of populations of invasive ants to supercolonies, as similar studies mostly rely on aggression behaviour and genetic analyses. We found that the three approaches revealed consistent but also complementary data and that both genetic and chemical variation within populations can be used as a powerful tool for the detection of founder effects. Still, microsatellites seem to be a more powerful tool to reconstruct introduction histories, as they moreover reveal the possible direction of spread (Figure 5). The cheapest and easiest of the three methods, behavioural observation of aggression, which can also be performed quickly in the field, was also quite informative, given that we and other studies showed that aggression levels between populations depend on the chemical profiles, which themselves are genetically based [35].

Our complementary approach revealed that all extant European populations of the invasive garden ant are probably derived from very few independent introductions out of the hitherto unknown native species range. Even if we do not yet know where the native populations of this new pest ant can be found, the development of highly variable microsatellite markers and the genetic 'barcode' of the introduced sites that we have obtained in this study will allow potential candidates for the native population from which introductions into Europe have occurred to be tested quickly. Our data indicate that most of the younger introductions of *L. neglectus *have likely arisen from other already established invasive populations. This implies that many more infestations of the invasive garden ant are likely to have taken place already, but have remained undiscovered owing to the usual lag phase for invasive species [3]. Many of these small and not yet well-established populations will likely have escaped notice because their negative effects on native communities are not currently apparent. However, recent decades have shown that established populations of *L. neglectus *do expand very fast [19,20,50] and are nearly impossible to eliminate [56]. We hope that our present study will contribute to the establishment of greater awareness to this pest ant, so that new infestations can be exterminated before they become damaging [57].

## Methods

### Study populations

*L. neglectus *was collected from 14 populations (Figure 1), representing a well-balanced subset of localities from the European distribution range of this species: Belgium (Ghent), France (Paris, Toulouse), Germany (Jena), Hungary (Budapest, Debrecen), Italy (Volterra), Poland (Warsaw), Spain (Bellaterra, Seva; both in the Barcelona area) and Turkey (Bayramiç, three separated populations in Edirne: 1, Sanayi Sitesi; 2, Selimiye Mosque; 3, Muammer Aksoy Caddesi). For each population, live workers from at least five randomly selected nests (minimum distance 20 m) were collected in 2003. Population boundaries were determined by mapping the presence of foraging workers on trees and shrubs. Sharp transitions were (almost) always found and were marked by GPS. These GPS measurements allowed us to estimate the approximate area in square kilometres for each population. Information about the year of discovery for each population was obtained from [20,31].

### Genetic analysis

#### DNA extraction and microsatellite polymerase chain reaction

DNA was extracted from 30 workers (5 nests and 6 workers/nest) per population, that is, from a total of 420 individuals. From each individual, the mesosoma was crushed in 200 μl of a 5% Chelex solution and boiled at 99°C for 15 min. For each sample six polymorphic nuclear microsatellite loci were amplified, three of them originally developed for *L. niger *[58], and three newly developed for *L. neglectus *(Table 2). The number of individuals that amplified at each locus ranged from 402 to 417. Polymerase chain reaction (PCR) conditions were as follows: initial denaturation 5 min at 95°C; 35 cycles of 30 s at 95°C, 30 s at the locus specific annealing temperature of 55/62°C, 60 s at 72°C; final elongation of 60 min at 72°C; reaction volume 20 μl; primer concentration 10 μM; Hybaid PCR Express Thermal Cycler. PCR products were run on 5% polyacrylamide gels using an ABI 377 automated sequencer with internal size standard (Rox500) and were analysed with GENESCAN 3.1 and GENOTYPER 3.1 (Applied Biosystems). Cross-amplification of all loci was tested for *L. alienus*, *L. austriacus*, *L. niger*, *L. turcicus *and *L. sakagamii*. All loci amplified under the same conditions as for *L. neglectus*, except in *L. austriacus *where locus Lng1 did not reliably amplify. Furthermore, all loci were found to be polymorphic in all tested species (although for *L. niger *this information does not exist for the three newly developed loci).

#### Genetic diversity and phylogenetic relationships

The genetic relationship between nestmate workers was assessed by measuring intra-nest regression relatedness using RELATEDNESS v. 5.0.8 [59] in all but the two populations in Spain (from which only a single nest had been sampled). Nests were weighted equally in this analysis. Allelic richness within populations (*k*') and pairwise genetic distances between populations (*F*_ST_) were calculated in FSTAT 2.9.3.2 [60] using 2000 permutations. A phylogram was created based on a distance matrix of allele frequency data (following the 'additive tree model') obtained from the six microsatellite loci using PHYLIP 3.6 [61]. Genetic distances for constructing the phylogram were calculated using Cavalli-Sforza chord measures, and bootstrap values were obtained by 2000 permutations. In addition, the Bayesian clustering program BAPS 2.0 [62] was used to group populations in clusters according to overall genetic similarity. We used populations without any sub-level as the level of sampling in these analyses. Estimations were performed across 50,000 iterations with 'thinning = 3' and a burn-in period of 10,000. The executed *P*-values give the probability of specific populations separating into one or more clusters of populations.

#### Detection of genetic bottlenecks

The populations were tested for the occurrence of genetic bottlenecks using both M RATIO [63] and BOTTLENECK 1.2.02 [55,64]. These two programs were chosen as they detect genetic bottlenecks without requiring a native reference population, which is unknown in *L. neglectus*. The two programs differ in that M RATIO is more likely to detect older bottleneck events than BOTTLENECK. M RATIO exploits the fact that allele distributions tend to shift after a genetic bottleneck and that the number of alleles is expected to decline faster than the overall range size of alleles. The program thus compares the ratio *M *(the number of alleles divided by the allele size range) to a distribution obtained by simulating 10,000 runs of a population at equilibrium. Based on earlier estimates of the effective population size *n*_e _of a *L. neglectus *population [24,65] and after comparing the size of this population with our data, we set *n*_e _= 5000. The mutation rate was set at *μ *= 10^-4^/locus/generation, which is the commonly used mutation rate for microsatellite loci in ants [66,67], resulting in a parameter setting of θ = 4*n*_e_*μ = 2. BOTTLENECK, on the other hand, detects divergences from allelic equilibrium in populations that have undergone a recent decrease in effective population size through estimating across 1000 permutations: (1) any reduction in genetic diversity, that is, a lower number of alleles than expected; and (2) any excess of heterozygosity per locus compared with an equilibrium population. In both software packages the two-phased model (TPM) was chosen as it provided the best fit for the evolution of microsatellite markers [55].

### Chemical analysis

#### Sampling and extraction

The cuticular hydrocarbon profiles of 68 nest samples from the 14 populations (3–7, mean 4.9 ± 1.1 nests per population) were analysed. Immediately after transportation of the live ants to the laboratory, five ants per nest were placed together in glass vials (Supelco; 1.8 ml), and stored at -20°C until solvent extraction. To extract cuticular compounds from the ants, pentane (50 μl) was added after the individuals had been transferred into small glass inserts (200 μl). After 10 min (during which the vials were vortexed three times) the ants were removed from the pentane and discarded. The vials were left open at room temperature until all of the pentane had evaporated. The dry extracts were kept frozen (-20°C) until they were analysed using GC-MS.

#### GC-MS

Shortly before GC-MS analysis, the samples were re-diluted in 20 μl of hexane. We injected 2 μl of each extract (split-less and via an auto-sampler) onto the GC-MS (Agilent Technologies 6890N coupled to a 5973N MSD) equipped with a HP-5 column (30 m × 0.25 mm, 0.25 μm film thickness). The initial oven temperature was set at 70°C. After 1 min the oven temperature was increased to 280°C at 20°C/min, after which it was increased further to 340°C at 2°C/min, and kept at this temperature for another 5 min. Other machine settings and characterisation of compounds followed the methods described by Lommelen et al [68].

#### Analysis and choice of compounds

Cuticular hydrocarbons (alkanes and mono-, di- and tri-methyl alkanes) were characterised using standard MS databases and diagnostic ions and by determining Kovats indices [69]. The position of double bonds in the alkenes was determined by derivatisation with di-methyl disulfide [70]. The methyl position in the methyl branched alkenes was first determined via a hydrogenation reaction with hydrogen and a palladium catalyst. The extracts contained both hydrocarbons and non-hydrocarbons (ketones, aldehydes and alcohols), but in the statistical analysis only the hydrocarbons were used, because other substances could be pheromones (e.g. alarm substances) rather than compounds used for nestmate recognition. For the same reason, n-undecane (C11), which occurred in some extracts, was excluded from the analysis, since it has been reported as an alarm substance from Dufour glands in two other *Lasius *species [71]. The final data set consisted of 24 remaining hydrocarbons, which varied in chain length between C31 and C35 (see Figure 3a and Additional file 2 for details).

#### Statistical hydrocarbon analysis

For all 68 nest samples, the area below each peak was integrated (CHEMSTATION, Agilent Technologies) and the relative proportions of the 24 hydrocarbon peaks were analysed using PCA in STATISTICA 6 (Statsoft) as in D'Ettorre and Heinze [72] revealing eight factors with eigenvalues over 0.7 explaining 82% of the total variance. A discriminant analysis based on these PCA factor scores was run with the 14 populations as a grouping variable. Mahalanobis distances between all pairwise populations were calculated as estimates for the chemical distances between populations (see Additional file 1). Mean Mahalanobis distances of all nests per population to the respective population mean (group centroid) were also calculated as an estimate for the within-population variation in cuticular hydrocarbon profiles. For each nest, the posterior probability of this nest to be correctly assigned to its original population from where it was sampled was determined. Nests were classified as mismatches in cases where the posterior probability was higher for an alternative population than the population it was sampled in (see Additional file 3).

### Behavioural assays

Within the first five weeks after the ants had been collected from the field, aggression tests were carried out in the laboratory between pairs of workers. To test whether single populations consisted of single supercolonies, the five sampled nests were tested in all pairwise combinations (one replicate each; for all but the two Spanish populations from which only a single nest could be sampled; total number of within-population between-nest aggression tests: *n *= 179). In addition, within-nest aggression tests were performed with two workers collected from identical nests (one replicate for each nest, that is, total *n *= 60). Aggression was never observed between any nests within the same population. We therefore chose one nest per population at random, with which we performed between-population tests. The 14 populations allowed for 91 independent pairwise combinations and each was tested in 7 replicates on average (range 3–12). As the Edirne 3 population behaved identically to the Edirne 1 population in all test trials we did not perform all replicates for Edirne 3. Taken together, a total of 579 aggression tests were carried out.

Five minutes before each aggression test, individual ants were removed from their rearing boxes and placed in individual plastic vials (diameter 2 cm, height 5 cm; fluon coated sides). The ants were then put into an 'arena' (Petri dish, 5.5 cm diameter; preliminary tests revealed that the outcome of any fight was independent of the order of putting the ants into the dish), and observed for 10 min at an ambient temperature of 22.5 ± 2°C following Giraud et al [34]. The frequency of six behaviours was recorded: ignorance behaviour (no interactive behaviour expressed when the two ants meet), antennation (one or both ants touch the other with their antennae), avoidance behaviour (one or both ants flee), gaster raising (aggressive behaviour often linked to the spraying of formic acid), biting and escalated fight. Encounters were classified as 'non-aggressive' when only ignorance and antennation behaviour occurred and as 'aggressive' when gaster raising, biting or fighting took place. The proportion of aggressive replicates per population pair was calculated as the number of aggressive replicates on the total number of replicates, revealing the behavioural distance between populations. In 1% of the encounters (7/579) only avoidance behaviour occurred in addition to ignorance and/or antennation. These encounters were excluded from the analysis as they could not be clearly assigned to either the aggressive or non-aggressive category.

### Correlations between geography, genetics, chemistry and behaviour

Partial Mantel correlation tests using distance matrices from inter-population geographic (in kilometres), genetic (as pairwise *F*_ST_), chemical (as Mahalanobis distances received from the discriminant analysis) and behavioural distance (as proportion of aggressive interactions) were carried out in FSTAT 2.9.3.2 using 2000 randomisations. Two-tailed *P*-values are reported.

## Authors' contributions

Ant populations were sampled by SC, JSP and LVU. Microsatellite primers were newly developed by DJCK and data were obtained by LVU. Cuticular hydrocarbon analyses were performed by FPD and SC. Aggression tests were carried out by SC and LVU. The study was designed by SC, JSP and JJB, the paper was written by these authors and LVU. All authors read and approved the manuscript.

## Supplementary Material

Additional file 1 **Population differentiation based on genetic, chemical, behavioural and geographic pairwise distances**. (a) Pairwise genetic distances (*F*_ST_) between sampled populations of *L. neglectus*, using the six microsatellite loci (Table 2). Bold figures indicate significance after multiple comparisons, *P *< 0.05. Order of populations after geography as in Figure 4. (b) Pairwise chemical (Mahalanobis) distances based on discriminant analysis of the cuticular hydrocarbon profiles between the sampled *L. neglectus *populations (see Figure 3 and Additional file 2). (c) Pairwise behavioural distance (aggression probabilities) between the sampled *L. neglectus *populations (Figure 4). (d) Pairwise geographic distances (in kilometres) between the study populations of *L. neglectus *(Figure 1). (e) Partial correlations between genetic, chemical, behavioural and geographic distance between the *L. neglectus *populations. Correlation coefficients (*r*_x,y.z_) are given for each plot. Mantel tests showed that correlation coefficients were only significant for behavioural distance versus geographic, genetic and chemical distance. Click here for file

Additional file 2 **Cuticular hydrocarbon compounds of *L. neglectus***. Peak number (see profile Figure 3(a)), short and full name of the 24 cuticular hydrocarbon compounds in the profile of *L. neglectus*. Click here for file

Additional file 3 **Correct and incorrect posterior assignment of nests to their population based on cuticular hydrocarbon profiles**. Posterior probabilities of each nest to be assigned to its original population in which it was sampled based on the chemical hydrocarbon profile. For cases in which posterior probabilities were higher for another population (bold), this posterior probability, as well as the identification of the other population, is given. Click here for file
