# Correction to: The introduction history of invasive garden ants in Europe: integrating genetic, chemical and behavioural approaches

**DOI:** 10.1186/s12915-018-0604-6

**Published:** 2018-10-30

**Authors:** Line V. Ugelvig, Falko P. Drijfhout, Daniel J. C. Kronauer, Jacobus J. Boomsma, Jes S. Pedersen, Sylvia Cremer

**Affiliations:** 1 Centre for Social Evolution, Department of Biology, University of Copenhagen, Universitetsparken 15, 2100 Copenhagen, Denmark; 2 Evolution, Genetics and Behaviour, Biology I, Institute of Zoology, University of Regensburg, 93040 Regensburg, Germany; 3 Chemical Ecology, School of Physical and Geographical Sciences, Keele University, Keele, Staffordshire ST5 5BG UK

## Correction

Reinvestigation of the raw data revealed an unfortunate error in Ugelvig et al. 2008 [1]. Our estimates of the area over which ant populations occurred erroneously entered diameters rather than radii in the ellipse area equation, which implied that population sizes were 4-fold overestimated except for one case (Warsaw) where we used a direct record of estimated area.

We here provide the corrected estimates in a new version of Table 1 and a new version of Fig. 5 in which relative population sizes were presented as diameters of the circles plotted. Moreover, we report the corrected statistical outcomes of the Pearson correlation tests between population size and age, chemical variation and allelic richness of populations. None of the conclusions in our paper is affected by this mistake, but correcting a 4-fold estimation error is important for future use of our data, because the new areas, for example, indicate that invasive populations of *Lasius neglectus* are detectable at smaller sizes than our previous estimates suggested.

Please see the correct figure (Fig. 5: Genetic diversity as a function of population age) and table (Table 1: Age and diversity of European Lasius neglectus populations) below.

**Fig. 5:**
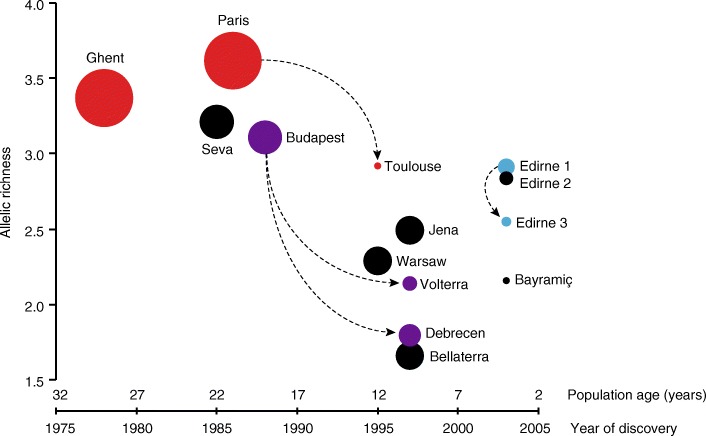
Genetic diversity as a function of population age. Mean allelic richness as a function of population age and population size (size of dots proportional to population area as given in Table 1) showing that allelic richness is generally lower in younger and smaller populations of *L. neglectus*. Arrows indicate likely genetic relations within genetic clusters, that is, older and more genetically diverse populations probably have given rise to younger populations. Populations are coloured according to the genetic clusters

**Table 1:** Age and diversity of European *Lasius neglectus* populations. Population name, country and year of discovery; *A*, population size in square kilometres; CHC var., within-population variation of cuticular hydrocarbon profiles; *P*, number of polymorphic loci (out of six microsatellite loci); *k’*, average allelic richness across all loci; *DH*/sd, standardised difference between the expected heterozygosity under mutation-drift equilibrium and observed heterozygosity; *M*, ratio between allele number and range. The latter two estimates are averages across polymorphic loci. Bold figures indicate significances in individual tests, whereas asterisks indicate significance levels after sequential Bonferroni correction for multiple tests: *P < 0.05; **P < 0.01

	Year	*A*	CHC var	*P*	*k’* ± SE	*DH*/sd ± SE	*M*
Ghent, Belgium	1978	0.143	10.797	4	3.37 ± 1.02	−0.23 ± 0.39	**0.576**
Seva, Spain	1985	0.050	8.413	4	3.21 ± 1.15	−0.23 ± 0.36	0.625
Paris, France	1986	0.141	12.538	5	3.62 ± 1.09	0.45 ± 0.37	0.742
Budapest, Hungary	1988	0.049	4.777	5	3.11 ± 0.77	−1.88 ± 0.71	**0.603**
Toulouse, France	1995	0.002	5.566	3	2.92 ± 0.97	−0.28 ± 0.81	0.560
Warsaw, Poland	1995	0.035	4.691	3	2.29 ± 0.64	0.43 ± 0.35	0.694
Bellaterra, Spain	1997	0.034	3.498	3	1.66 ± 0.33	0.34 ± 0.73	0.756
Debrecen, Hungary	1997	0.021	3.338	2	1.80 ± 0.52	−0.94 ± 1.33	0.563
Jena, Germany	1997	0.035	5.796	5	2.49 ± 0.42	1.06* ± 0.15	0.503*
Volterra, Italy	1997	0.009	3.141	3	2.14 ± 0.58	−0.86 ± 0.43	0.720
Bayramiç, Turkey	2003	0.002	7.730	3	2.16 ± 0.54	0.35 ± 0.20	0.338**
Edirne1, Turkey	2003	0.012	8.545	4	2.91 ± 0.83	0.19 ± 0.51	0.600
Edirne2, Turkey	2003	0.008	2.405	3	2.84 ± 0.94	−1.28 ± 1.36	**0.551**
Edirne3, Turkey	2003	0.004	7.100	3	2.55 ± 0.77	−0.25 ± 0.60	0.419*

Furthermore, three sentences in the Result section “Age and diversity of populations” read as follows in the original manuscript:

*“*As all populations were assumed to have started with a small founder group of a single or very few nests, the present size of a population was also expected to be an indicator of age and both were indeed highly correlated (Pearson correlation, *n* = 14, *r* = − 0.838, *P* = 0.0002; see Table 1 for details). We determined the chemical variation within populations (that is, the mean chemical distance of nests to their group centroid; Table 1) and found that the chemical variation was significantly negatively correlated with the discovery date of the populations (Pearson correlation, *n* = 14, *r* = − 0.513, *P* = 0.060) and positively correlated with present population size (*r* = 0.688, *P* = 0.006). We found similar results for correlations of the allelic richness for each population based on the microsatellite analysis, with both year of detection (Fig. 5; Pearson correlation, *n* = 14, *r* = − 0.600, *P* = 0.023) and population size (*r* = 0.632, *P* = 0.0154), as well as a high correlation between allelic richness and chemical within-population variation (*r* = 0.745, *P* = 0.002).”

After correction, the paragraph reads as follows. It contains the correlation results based on the corrected population sizes with population age, chemical variation and allelic richness. Moreover, we removed the word ‘significantly’ in one of the sentences because one of correlations (*P* = 0.06) is only marginally significant, whilst the other speaks for itself, remaining highly significant after recalculation.

*“*As all populations were assumed to have started with a small founder group of a single or very few nests, the present size of a population was also expected to be an indicator of age and both were indeed highly correlated (Pearson correlation, *n* = 14, *r* = − 0.849, *P* = 0.0001; see Table 1 for details). We determined the chemical variation within populations (that is, the mean chemical distance of nests to their group centroid; Table 1) and found that the chemical variation was negatively correlated with the discovery date of the populations (Pearson correlation, *n* = 14, *r* = − 0.513, *P* = 0.060) and positively correlated with present population size (*r* = 0.702, *P* = 0.005). We found similar results for correlations of the allelic richness for each population based on the microsatellite analysis, with both year of detection (Fig. 5; Pearson correlation, *n* = 14, *r* = − 0.600, *P* = 0.023) and population size (*r* = 0.616, *P* = 0.0190), as well as a high correlation between allelic richness and chemical within-population variation (*r* = 0.745, *P* = 0.002).”

The legends for Table 1 and Fig. 5 remain unchanged.
