# Supplementary material for: The introduction history of invasive garden ants in Europe: Integrating genetic, chemical and behavioural approaches
Source: BMC Biol. 2008 Feb 26;6:11. doi: 10.1186/1741-7007-6-11 (PMC2292682; doi:10.1186/1741-7007-6-11)

Additional files

Additional file 1. Population differentiation based on genetic, chemical, behavioural and geographic pairwise distances.

a) Pairwise genetic distances (F_{ST}) between sampled populations of *L. neglectus*, using the six microsatellite loci (Table 2). Bold figures indicate significance after multiple comparisons, $P < 0.05$. Order of populations after geography as in Figure 4.

	Jena	Warsaw	Edirne 2	Edirne 1	Edirne 3	Bayramiç	Debrecen	Budapest	Volterra	Seva	Bellaterra	Ghent	Paris	Toulouse
Jena	—													
Warsaw	**0.352**	—												
Edirne 2	**0.373**	**0.396**	—											
Edirne 1	**0.383**	**0.239**	**0.338**	—										
Edirne 3	**0.431**	**0.334**	**0.425**	0.021	—									
Bayramiç	**0.383**	**0.318**	**0.415**	**0.342**	**0.409**	—								
Debrecen	**0.511**	**0.468**	**0.497**	**0.448**	**0.542**	**0.568**	—							
Budapest	**0.431**	**0.352**	**0.390**	**0.348**	**0.445**	**0.474**	0.019	—						
Volterra	**0.464**	**0.404**	**0.447**	**0.399**	**0.491**	**0.501**	0.043	0.018	—					
Seva	**0.366**	**0.108**	**0.347**	**0.217**	**0.324**	**0.312**	**0.350**	**0.242**	**0.298**	—				
Bellaterra	**0.462**	**0.368**	**0.551**	**0.466**	**0.549**	**0.441**	**0.549**	**0.422**	**0.474**	**0.271**	—			
Ghent	**0.261**	**0.167**	**0.284**	**0.220**	**0.307**	**0.328**	**0.335**	**0.217**	**0.246**	**0.139**	**0.373**	—		
Paris	**0.212**	**0.165**	**0.228**	**0.176**	**0.249**	**0.284**	**0.321**	**0.220**	**0.258**	**0.156**	**0.371**	**0.031**	—	
Toulouse	**0.261**	**0.216**	**0.322**	**0.267**	**0.349**	**0.361**	**0.363**	**0.235**	**0.270**	**0.189**	**0.374**	**0.033**	**0.044**	—

b) Pairwise chemical (Mahalanobis) distances based on discriminant analysis of the cuticular hydrocarbon profiles between the sampled *L. neglectus* populations (Figure 3, Additional File 2).

	Jena	Warsaw	Edirne 2	Edirne 1	Edirne 3	Bayramiç	Debrecen	Budapest	Volterra	Seva	Bellaterra	Ghent	Paris	Toulouse
Jena	—													
Warsaw	173.595	—												
Edirne 2	33.979	92.420	—											
Edirne 1	9.552	140.613	18.201	—										
Edirne 3	18.581	149.683	17.890	8.220	—									
Bayramiç	35.793	223.794	56.509	28.214	15.715	—								
Debrecen	21.854	100.649	9.717	16.584	29.181	63.677	—							
Budapest	34.678	140.078	28.834	25.275	50.795	88.598	13.354	—						
Volterra	25.644	155.667	56.398	27.440	49.608	68.254	29.940	46.767	—					
Seva	29.804	146.094	30.896	21.646	31.089	46.918	17.047	29.688	21.712	—				
Bellaterra	20.631	103.107	20.772	17.348	36.459	71.430	3.606	14.575	16.121	14.543	—			
Ghent	11.982	235.529	65.184	23.861	30.436	28.437	47.719	59.601	30.338	28.410	41.879	—		
Paris	25.614	111.832	29.515	29.610	47.773	76.471	10.172	18.716	45.017	34.589	13.342	53.433	—	
Toulouse	26.826	111.445	28.222	23.418	45.113	81.553	7.377	12.738	18.391	13.060	1.783	44.508	16.841	—

c) Pairwise behavioural distance (aggression probabilities) between the sampled *L. neglectus* populations (Figure 4).

Jena	0.00													
Warsaw	1.00	0.00												
Edirne 2	1.00	1.00	0.00											
Edirne 1	0.60	1.00	0.55	0.00										
Edirne 3	0.60	1.00	0.55	0.00	0.00									
Bayramiç	1.00	1.00	1.00	0.33	0.33	0.00								
Debrecen	0.80	0.80	0.67	0.58	0.58	0.67	0.00							
Budapest	0.60	0.80	1.00	0.58	0.58	0.33	0.30	0.00						
Volterra	0.00	0.78	1.00	0.70	0.70	0.33	0.20	0.20	0.00					
Seva	0.50	0.40	1.00	0.60	0.60	0.67	0.11	0.00	0.00	0.00				
Bellaterra	0.40	0.70	0.67	0.64	0.64	0.00	0.70	0.80	0.30	0.70	0.00			
Ghent	0.44	0.89	1.00	0.60	0.60	1.00	0.22	0.00	0.25	0.00	0.44	0.00		
Paris	0.80	0.67	0.67	0.73	0.73	0.67	0.30	0.00	0.20	0.00	0.40	0.11	0.00	
Toulouse	0.00	0.60	1.00	0.60	0.60	0.67	0.30	0.20	0.00	0.00	0.44	0.00	0.00	0.00
	Jena	Warsaw	Edirne 2	Edirne 1	Edirne 3	Bayramiç	Debrecen	Budapest	Volterra	Seva	Bellaterra	Ghent	Paris	Toulouse

d) Pairwise geographic distances (in km) between the study populations of *L. neglectus* (Figure 1).

Jena	—													
Warsaw	825	—												
Edirne 2	1648	1267	—											
Edirne 1	1651	1270	2	—										
Edirne 3	1651	1270	3	1	—									
Bayramiç	1788	1463	208	206	206	—								
Debrecen	943	525	778	780	781	960	—							
Budapest	748	560	912	914	915	1071	224	—						
Volterra	839	1315	1364	1366	1367	1412	1034	834	—					
Seva	1289	1984	2089	2091	2091	2105	1781	1570	759	—				
Bellaterra	1328	2019	2104	2106	2107	2117	1809	1599	783	41	—			
Ghent	677	1494	2224	2226	2227	2332	1589	1376	1048	1030	1069	—		
Paris	850	1672	2242	2244	2245	2327	1683	1460	955	763	801	293	—	
Toulouse	1193	1938	2169	2171	2172	2203	1790	1570	809	206	238	851	572	—
	Jena	Warsaw	Edirne 2	Edirne 1	Edirne 3	Bayramiç	Debrecen	Budapest	Volterra	Seva	Bellaterra	Ghent	Paris	Toulouse

e) Partial correlations between genetic, chemical, behavioural and geographic distance between the *L. neglectus* populations. Correlation coefficients ($r_{x,y.z}$) are given for each plot. Mantel tests showed that correlation coefficients were only significant for behavioural distance vs. geographic, genetic and chemical distance.

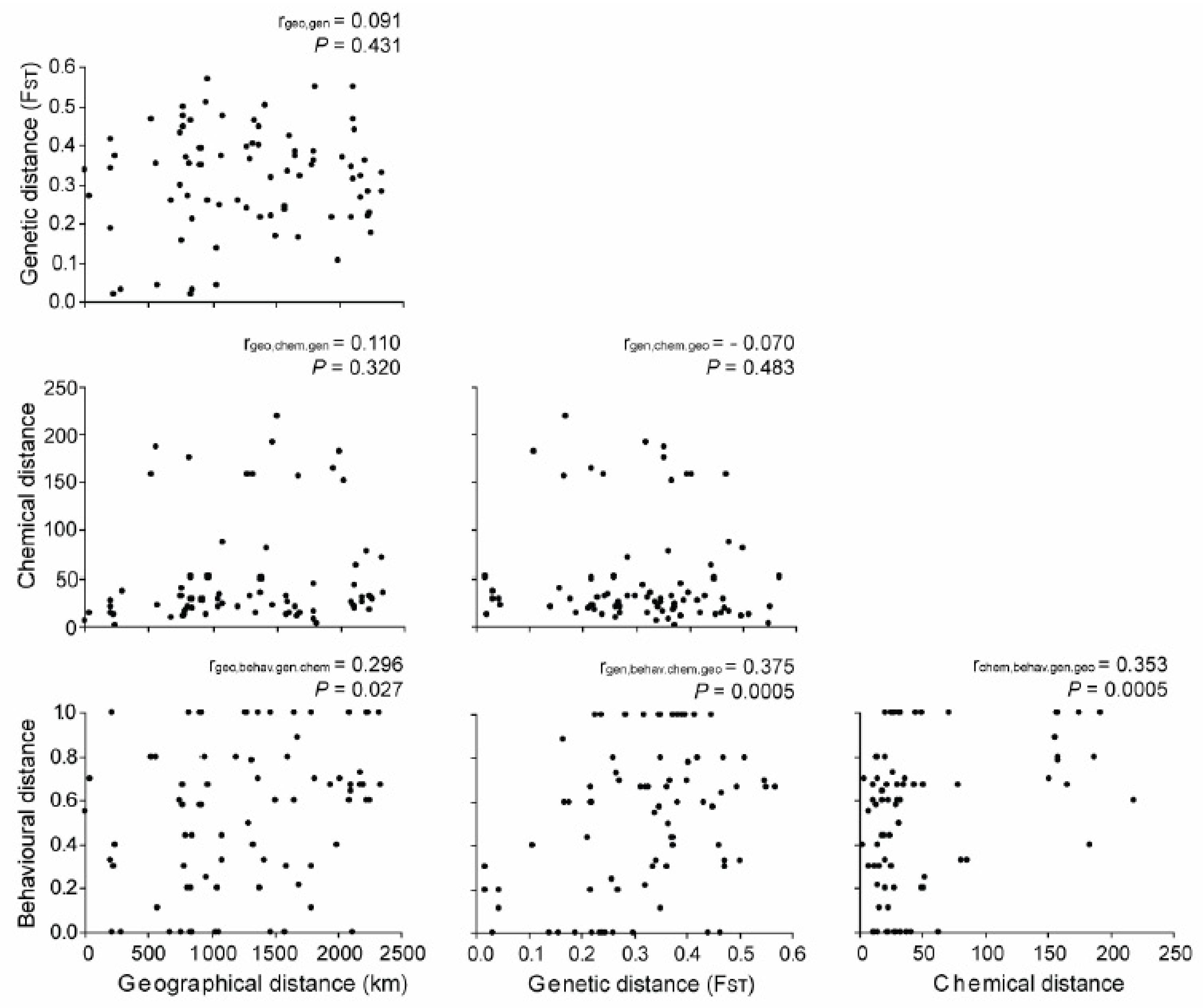

Supplement: Additional file 1 — Population differentiation based on genetic, chemical, behavioural and geographic pairwise distances. (a) Pairwise genetic distances (FST) between sampled populations of L. neglectus, using the six microsatellite loci (Table 2). Bold figures indicate significance after multiple comparisons, P < 0.05. Order of populations after geography as in Figure 4. (b) Pairwise chemical (Mahalanobis) distances based on discriminant analysis of the cuticular hydrocarbon profiles between the sampled L. neglectus populations (see Figure 3 and Additional file 2). (c) Pairwise behavioural distance (aggression probabilities) between the sampled L. neglectus populations (Figure 4). (d) Pairwise geographic distances (in kilometres) between the study populations of L. neglectus (Figure 1). (e) Partial correlations between genetic, chemical, behavioural and geographic distance between the L. neglectus populations. Correlation coefficients (rx,y.z) are given for each plot. Mantel tests showed that correlation coefficients were only significant for behavioural distance versus geographic, genetic and chemical distance. [file 1741-7007-6-11-S1.pdf]
