# Supplementary material for: The introduction history of invasive garden ants in Europe: Integrating genetic, chemical and behavioural approaches
Source: BMC Biol. 2008 Feb 26;6:11. doi: 10.1186/1741-7007-6-11 (PMC2292682; doi:10.1186/1741-7007-6-11)
Supplement: Additional file 2 — Cuticular hydrocarbon compounds of L. neglectus. Peak number (see profile Figure 3(a)), short and full name of the 24 cuticular hydrocarbon compounds in the profile of L. neglectus. [file 1741-7007-6-11-S2.pdf]

**Additional file 2. Cuticular hydrocarbon compounds of *Lasius neglectus*.**

Peak number (see profile Figure 3a), short and full name of the 24 cuticular hydrocarbon compounds in the profile of *L. neglectus*.

| Peak number | Short compound name | Full compound name |
|---|---|---|
| 1 | 11C31:1 | hentriacont-11-ene |
| 2 | C31 | hentriacontane |
| 3 | 13MeC31 | 13Methylhentriacontane |
| 4 | 3MeC31 | 3Methylhentriacontane |
| 5 | C33:2 | tritriacontadiene |
| 6 | C33:2 | tritriacontadiene |
| 7 | C33:2 | tritriacontadiene |
| 8 | 12C33:1 + 13C33:1 | tritriacont-12-ene + tritriacont-13-ene |
| 9 | 10C33:1 + 11C33:1 | tritriacont-10-ene + tritriacont-11-ene |
| 10 | 7C33:1 | tritriacont-7-ene |
| 11 | 13Me21C33:1 + 13Me23C33:1 | 13Methyltritriacont-21-ene + 13Methyltritriacont-23-ene |
| 12 | 13MeC33 + 15MeC33 | 13Methyltritriacontane + 15Methyltritriacontane |
| 13 | 3Me21C33:1 | 3Methyltritriacont-21-ene |
| 14 | 3Me23C33:1 | 3Methyltritriacont-23-ene |
| 15 | 10,23diMeC33 | 10,23Dimethyltritriacontane |
| 16 | 3MeC33 + 5,15diMeC33 | 3Methyltritriacontane + 5,15Dimethyltritriacontane |
| 17 | 12,14,22Me21C34:1 + 12,14,22Me23C34:1 | 12,14,22Methyltetratriacont-21-ene + 12,14,22Methyltetratriacont-23-ene |
| 18 | C35:2 | pentatriacontadiene |
| 19 | C35:2 | pentatriacontadiene |
| 20 | C35:2 | pentatriacontadiene |
| 21 | 21C35:1 | pentatriacont-21-ene |
| 22 | 23C35:1 | pentatriacont-23-ene |
| 23 | 13,15Me21C35:1 + 13,15Me23C35:1 | 13,15Methylpentatriacont-21-ene + 13,15Methylpentatriacont-23-ene |
| 24 | 13MeC35 + 15MeC35 | 13Methylpentatriacontane + 15Methylpentatriacontane |
