# Supplementary material for: The introduction history of invasive garden ants in Europe: Integrating genetic, chemical and behavioural approaches
Source: BMC Biol. 2008 Feb 26;6:11. doi: 10.1186/1741-7007-6-11 (PMC2292682; doi:10.1186/1741-7007-6-11)
Supplement: Additional file 3 — Correct and incorrect posterior assignment of nests to their population based on cuticular hydrocarbon profiles. Posterior probabilities of each nest to be assigned to its original population in which it was sampled based on the chemical hydrocarbon profile. For cases in which posterior probabilities were higher for another population (bold), this posterior probability, as well as the identification of the other population, is given. [file 1741-7007-6-11-S3.pdf]

Additional file 3. Correct and incorrect posterior assignment of nests to their population based on cuticular hydrocarbon profiles.

Posterior probabilities of each nest to be assigned to its original population in which it was sampled based on the chemical hydrocarbon profile. Cases, in which posterior probabilities were higher for another population (bold), this posterior probability, as well as the identification of the other population is given.

		Posterior probability for		
Original population	Nest number	Original population	Alternative population	Alternative population
Ghent	1	0.99		
	**2**	**0.37**	**0.62**	**Jena**
	3	1.00		
	4	0.87		
	5	1.00		
Seva	1	0.98		
	2	0.99		
	3	0.99		
	4	0.91		
	5	1.00		
Paris	**1**	**0.00**	**0.38**	**Bellaterra**
	2	0.50		
	3	1.00		
	4	0.96		
Budapest	**1**	**0.19**	**0.34**	**Toulouse**
	2	0.97		
	3	1.00		
Toulouse	1	0.87		
	2	0.67		
	3	0.40		
	4	0.38		
	**5**	**0.31**	**0.38**	**Volterra**
Warsaw	1	1.00		
	2	1.00		
	3	1.00		
	4	1.00		
	5	1.00		
Bellaterra	**1**	**0.34**	**0.60**	**Toulouse**
	**2**	**0.20**	**0.52**	**Volterra**
	**3**	**0.03**	**0.57**	**Debrecen**
	4	0.55		
	5	0.61		
Debrecen	**1**	**0.13**	**0.45**	**Toulouse**
	2	0.68		
	3	0.77		
	4	0.49		
	5	0.63		

Jena	1	0.91		
	2	0.96		
	**3**	**0.36**	**0.62**	**Edirne1**
	4	0.99		
	5	0.83		
Volterra	1	0.94		
	2	1.00		
	3	0.56		
Bayramiç	**1**	**0.00**	**0.77**	**Ghent**
	2	1.00		
	3	1.00		
	4	0.90		
	5	0.99		
Edirne1	1	0.87		
	2	0.89		
	3	0.60		
	4	0.83		
	5	0.80		
	**6**	**0.18**	**0.70**	**Budapest**
	7	0.74		
Edirne2	1	0.65		
	2	0.92		
	3	0.86		
	4	0.94		
	5	0.99		
	6	0.97		
Edirne3	1	0.95		
	2	0.78		
	3	0.50		
	4	0.97		
	5	0.59		
